# The role of non–COVID-specific and COVID-specific factors in predicting a shift in willingness to vaccinate: A panel study

**DOI:** 10.1073/pnas.2112266118

**Published:** 2021-12-20

**Authors:** Kathleen Hall Jamieson, Daniel Romer, Patrick E. Jamieson, Kenneth M. Winneg, Josh Pasek

**Affiliations:** ^a^Annenberg Public Policy Center, University of Pennsylvania, Philadelphia, PA 19104;; ^b^Department of Communication and Media, University of Michigan, Ann Arbor, MI 48109

**Keywords:** COVID-19, vaccination hesitancy, trust in health experts, COVID conspiracy beliefs, media reliance

## Abstract

Although declines in intent to vaccinate had been identified in international surveys conducted between June and October 2020, including in the United States, some individuals in the United States who previously expressed reluctance said, in spring 2021, that they were willing to vaccinate. That change raised the following questions: What factors predicted an increased willingness to inoculate against COVID-19? And, to what extent was the change driven by COVID-specific factors, such as personal worry about the disease and COVID-specific misinformation, and to what extent by background (non–COVID-specific) factors, such as trust in medical authorities, accurate/inaccurate information about vaccination, vaccination history, and patterns of media reliance? This panel study of more than 8,000 individuals found that trust in health authorities anchored acceptance of vaccination and that knowledge about vaccination, flu vaccination history, and patterns of media reliance played a more prominent role in shifting individuals from vaccination hesitance to acceptance than COVID-specific factors. COVID-specific conspiracy beliefs did play a role, although a lesser one. These findings underscore the need to reinforce trust in health experts, facilitate community engagement with them, and preemptively communicate the benefits and safety record of authorized vaccines. The findings suggest, as well, the need to identify and deploy messaging able to undercut health-related conspiracy beliefs when they begin circulating.

## Significance

In communities that remain below the immunity threshold needed to blunt COVID-19’s spread, SARS-CoV-2 has a greater chance of mutating to evade vaccines. This study underscores the central role of trust and knowledge in increasing the likelihood of vaccinating. Trust in scientific institutions and spokespersons anchors time 1 vaccination intentions and knowledge affects them at both times 1 and 2. These background (non–COVID-specific) factors as well as flu vaccination history and patterns of media reliance played a more prominent role in shifting individuals from vaccination hesitance to acceptance than did COVID-specific ones. The study underscores the need for ongoing community engagement and trust building, proactive communication about vaccination, motivating vaccination against seasonal flu, and deploying science-consistent, provaccination voices across media.

In late April 2021, public health officials expressed concern that many communities throughout the United States were not on track to reach the level of immunity required to halt the replication and hence possible additional mutation of severe acute respiratory syndrome coronavirus 2 (SARS-CoV-2) (1). Faced with lagging demand, vaccines were going unused (2), and vaccine sites were closing (3). Because the rate of uptake continued to slow, the nation fell short of the vaccination goal set by the incumbent Biden administration of 70% of the eligible population at least partially vaccinated by July 4, 2021 (4).

Although declines in intent to vaccinate had been identified in international surveys conducted between June and October 2020, including in the United States (5), by spring 2021, some individuals in the United States who previously expressed reluctance had, in fact, rolled up their sleeves. An April 2021 CBS News poll that found hesitance in 4 in 10 concluded, nonetheless, that the percentage who expressed reluctance had decreased over time (6). This changing receptivity to vaccination raised the following question: What factors predicted an increased willingness to inoculate against COVID-19? A US study that found such an increase from mid-October 2020 to the end of March 2021 associated the change with increased public trust in vaccine development and in the governmental approval process (7).

Past research has explored other factors that predict willingness to accept a COVID-19 vaccine as well. Some, such as education, are demographic. Others are COVID specific, including a positive attitude toward the COVID-19 vaccines, worry about COVID-19 infection, accurate beliefs about the disease, and rejection of COVID-specific conspiracy beliefs. Non–COVID-specific ones include past receipt of the flu vaccine, accurate vaccination beliefs, and mainstream media reliance (8, 9). And, because there is ample evidence that Democrats are more likely than Republicans to take a COVID-19 vaccine (10), the possibility that partisanship elicited different vaccination intentions among Republicans and Democrats before and after the election deserves scrutiny.

**Fig. 1.**
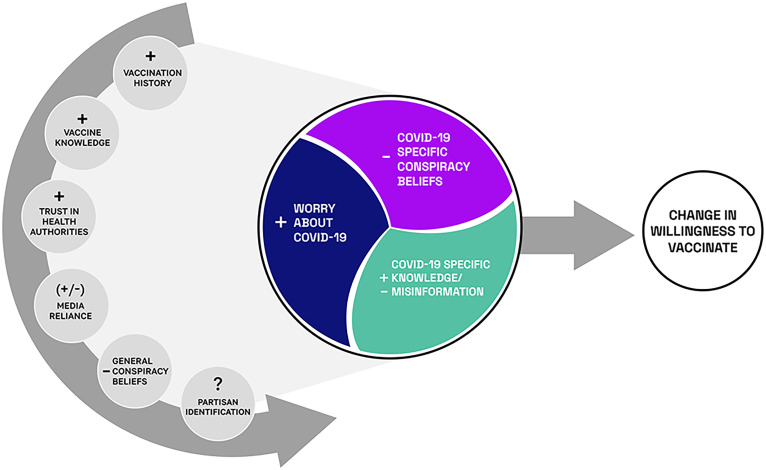
Model linking non–COVID-specific background variables and COVID-specific ones with change in vaccination intention.

**Fig. 2.**
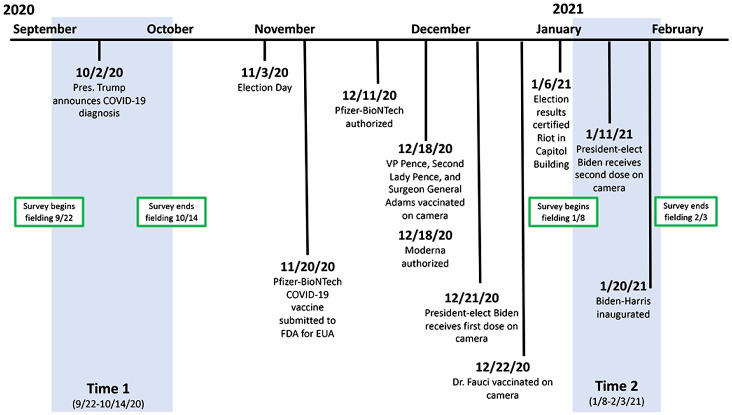
Timeline of panel survey and vaccination-relevant events.

Unpacking the factors associated with changing vaccination intentions is complicated. Strong attitudes are resistant to change, are stable, and influence both cognition and action (11). If key factors facilitating vaccination are non–COVID specific, an exclusive focus on COVID-specific ones would be unlikely to facilitate change in intention. At the same time, panel data are necessary to identify the kinds of individuals susceptible to persuasive messaging. To these ends, our model provides a framework for determining whether a person’s opposition to COVID vaccination will be durable or not (Fig. 1). It does so by disentangling factors that distinguish those who, in late September/early October 2020, were reluctant but, in January and early February 2021, expressed willingness to take the COVID-19 vaccine (Fig. 2). Our dependent variable is a positive change in disposition to vaccinate.

## Hypotheses

We anticipated that the background factors in the non–COVID-specific left half-circle in Fig. 1 would be primary determinants of a changed willingness to vaccinate and that the COVID-specific ones in the circle at the center of the model would exert a smaller impact. (See *SI Appendix* for questions asked.)

## The Panel

**Fig. 3.**
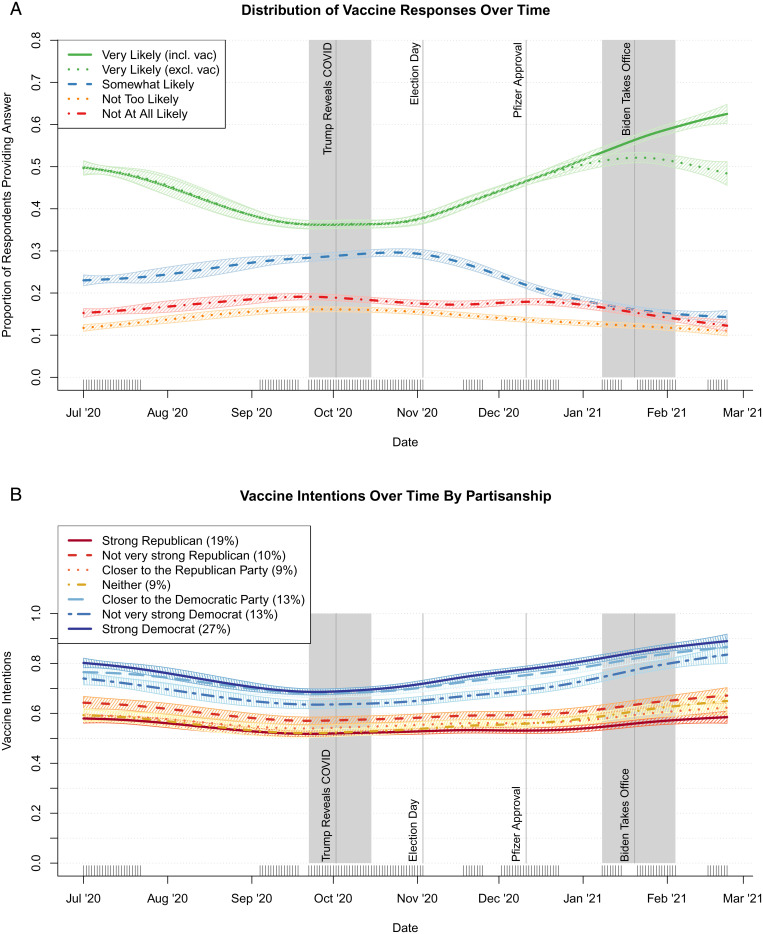
Trends in vaccination intention answers over time (*A*) and mean levels of vaccination intentions among individuals in each partisan group (*B*) based on GAMs (key waves shown in gray).

**Fig. 4.**
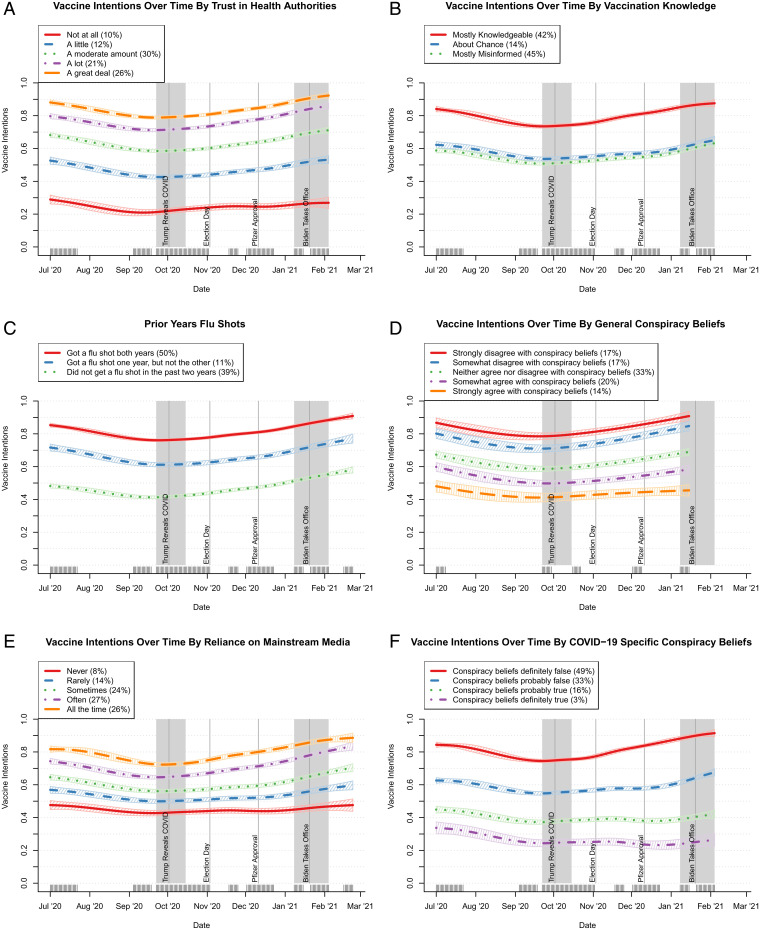
Trends in mean vaccination intentions by key variables estimated using GAMs (key waves shown in gray). Vaccine intentions over time by (*A*) trust in health authorities, (*B*) vaccination knowledge, (*C*) prior year flu shots, (*D*) general conspiracy beliefs, (*E*) reliance on mainstream media, and (*F*) COVID-19–specific conspiracy beliefs.

Data for the current study come from probability-based samples of Americans drawn in five states—Florida, Michigan, Ohio, Pennsylvania, and Wisconsin—as part of the 2020 Annenberg Institutions of Democracy (IOD) Election Study. Using address-based sampling, the survey firm SSRS recruited the panelists, who were surveyed between April of 2020 and March of 2021. Our analyses examine their answers about vaccination intentions in waves 3 (July of 2020), 5 (August/September of 2020), 6 (September/October of 2020), 7 (October/November of 2020), 8 (November of 2020), 9 (December of 2020), 10 (January/February of 2021), and 11 (February of 2021) of the larger study. Data from 11,017 individuals who answered these questions in at least one wave were used to construct estimates of trends in intentions by demographic groups (Figs. 3 and 4). To understand the factors associated with changing intentions over time, we focus on the answers of 8,496 respondents who completed these items in both waves 6 and 10 of the study (hereafter: time 1 and time 2). These waves were selected because they reflected the nadir of vaccination intentions and the last full wave of data collection (wave 11 was only asked of a random subset of panelists). Focusing on changes following the low point of vaccination intentions maximized our ability to identify the individual attributes that were associated with changes. The period between time 1 and time 2 includes the announcement that the trials of the Moderna and Pfizer vaccines were successful; Emergency Use Authorization (EUA) of these vaccines by the Food and Drug Administration (FDA); and the election, certification, and inauguration of President Joe Biden and Vice President Kamala Harris. The Pfizer-BioNTech COVID-19 vaccine was submitted to the FDA for EUA on November 20 and authorized on December 11, 2020. The Moderna vaccine received its FDA EUA on December 19, 2020.

## Background Factors

### Vaccination History.

Because prior behavior predicts future behavior (12), we anticipated that one’s vaccination history would predict a change in the likelihood of getting a COVID-19 vaccine. In particular, since those who had been vaccinated against flu in the past have been found to be more willing to take the COVID vaccine (13, 14), we expected vaccination against flu to predict such a change.

### Vaccination Knowledge.

Pre-COVID research found that the mistaken belief in a measles, mumps, and rubella (MMR) vaccine–autism link increased hesitancy about the MMR vaccine (15) and affected willingness to take a prospective vaccine unrelated to measles (16). We predicted that vaccination knowledge would increase one’s disposition to vaccinate, while being misinformed about vaccination in general and the effects of the MMR vaccine in particular would anchor reluctance.

### Trust.

Because trust in science and scientific authorities has been associated with vaccination acceptance (17) and, in past pandemics (including SARS, H5N1, and H1N1), has been associated with a willingness to take protective action (18) and because low trust in the governmental approval process has been associated with a reduced likelihood of taking the COVID vaccine (7, 19–21), we hypothesized that higher levels of trust in health authorities would increase the likelihood of shifting from vaccine reluctance to acceptance.

### Media Reliance.

Patterns of media exposure have been associated with holding misinformed views of vaccination in general (22) and, in particular, with holding COVID-related conspiracy beliefs which, in turn, is associated with a reduced intention to vaccinate (8). As a result, we hypothesized that reliance on conservative or social media would predict a lower likelihood of increased willingness to vaccinate and that reliance on mainstream media, including mainstream print, would be associated with an increased willingness to vaccinate.

### General Conspiracy Thinking/Beliefs.

We predicted that rejection of general conspiracy thinking/beliefs would increase the likelihood of shifting to a provaccination position, because research conducted prior to the COVID-19 pandemic found that those inclined to conspiratorial thinking were more likely to hold antivaccination positions (23) and to prefer alternatives to the traditional biomedical therapies endorsed by established evidence-based medicine and institutional medical authorities (24–26).

### Partisan Identification Research Questions.

Political parties and their leaders provide cues to their supporters that can shape attitudes and behaviors (27–29). Partisan differences in preventive behaviors, including masking (30–32) and physical distancing (33, 34), were evident during the pandemic. Because the presidential campaign contained vaccination-related cues from the 45th president of the United States and Democratic operatives, we treated the following as a research question: Were Democrats more accepting of vaccination and Republicans less so after the election of Joe Biden?

On the one hand, Trump championed vaccine development and took deserved credit for the role that his “Operation Warp Speed” played in incentivizing the development of COVID-19 vaccines. On the other hand, he failed to publicize his own vaccination and that of the first lady when both occurred in January before he left the presidency. By contrast, prominent Democrats not only expressed doubts about Trump’s confident assertions that a safe and effective vaccine would be in the offing by Election Day but also raised the possibility that he would sacrifice vaccine safety to achieve electoral advantage. “If @realDonaldTrump fast tracks a vaccine, bypassing critical safety steps so he can announce it before the election, who will have confidence in taking it?” tweeted former Obama aide David Axelrod in August 2020 (35). “If public health professionals, if Dr. Fauci, if the doctors tell us that we should take it, I’ll be the first in line to take it, absolutely,” Democratic Vice Presidential nominee Kamala Harris declared in the 2020 general election debate with Vice President Mike Pence. “But if Donald Trump tells us that we should take it, I’m not taking it.” “[P]lease stop undermining confidence in a vaccine,” responded her Republican counterpart (36). In early December, Biden set, as his goal, “at least 100 million Covid vaccine shots into the arms of the American people” during his first 100 d in office (37).

## The COVID-Specific Factors

Because a pre–COVID-19 metaanalysis (38) and studies during the COVID-19 pandemic found an association between perceived risk or threat of infection and willingness to get vaccinated (16, 18, 19, 21, 39–44), we included worry about getting COVID-19 in the model. We also included specific misinformation about the COVID-19 pandemic and the vaccine against it (45) and COVID-specific conspiracy theories (9), because each has been associated with reduced intention to take the COVID-19 vaccine.

### Findings.

The proportion intending to vaccinate changed notably over time, with those saying they were very likely to do so dropping from around half of respondents in July of 2020 to slightly more than a third in late September and early October, before rebounding in January/early February. Fig. 3*A* shows splines from generalized additive models (GAMS) estimating the prevalence of individuals responding, over time, that they were very likely, somewhat likely, not too likely, or not at all likely to vaccinate.* In total, 11.5% of weighted respondents reported a diminished intention to vaccinate compared to 33.6% who increased their willingness to do so (*SI Appendix*, Table S2). But these aggregate changes mask varying trends in intentions across population subgroups.

Background (non–COVID-specific factors) that were related to shifts in vaccination intention are shown in Fig. 4. As the figure indicates, those who expressed the greatest trust in public health authorities were, on average, far more willing to vaccinate than those who expressed less trust (Fig. 4*A*). Respondents who provided accurate answers to questions about general vaccine knowledge were also more prone to vaccinate than those who held misperceptions (Fig. 4*B*). These differences were largely steady over time. Changed intentions also were associated with prior flu vaccination history, acceptance of general conspiracy theories as well as coronavirus-specific ones, and patterns of media use (Fig. 4 *C*–*F*).

**Fig. 5.**
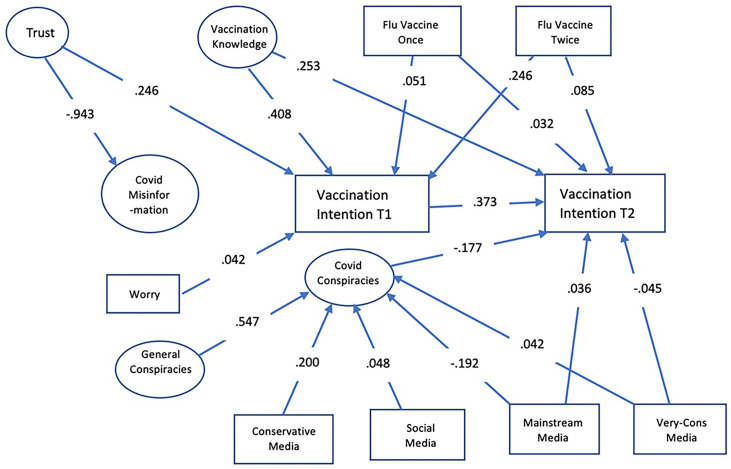
Standardized paths in SEM of predictors of change in vaccination intention. All paths were within 99% CIs. Paths for demographic and political controls are not shown (Table 1 and *SI Appendix*, Table S3).

To assess whether these factors (along with additional covariates and COVID-specific predictors shown in *SI Appendix*, Fig. S1) were related to intentions, we isolated the unique contributions of each by predicting changes between the nadir of vaccination intentions in late September/early October (time 1) and the subsequent increase in January/early February (time 2) using a structural equation model (SEM). Structural equation modeling allowed us to see how non–COVID-specific factors predicted COVID-specific ones and how both types of measures related to changing intentions to vaccinate, while controlling for the other variables in the model (shown in Fig. 5).

Table 1 presents the standardized coefficients and associated CIs for the SEM. The first column contains the predictors of vaccination at time 1 (September 22 to October 14, 2020), while the second has the predictors of change at time 2 (January 8 to February 3, 2021). The correlations with vaccination intention at time 1 are in the third column. The major components of the path model are displayed in Fig. 5.

**Table 1.** Standardized parameters and 99% CIs of vaccination intentions at time 1 and then prospectively for change at time 2

Predictor wave 1	Direct predictors of vaccination intention time 1	Direct predictors of vaccination intention change	Correlates of vaccination intention time 1
Trust in health authorities	0.246 (0.183, 0.307)		0.460 (0.426, 0.488)
COVID-specific misinformation			−0.461 (−0.493, −0.431)
COVID-specific conspiracy beliefs		−0.177 (−0.221, −0.131)	−0.427 (−0.452, −0.398)
General conspiracy thinking/beliefs			−0.365 (−0.412, −0.320)
Vaccination knowledge	0.408 (0.363, 0.459)	0.253 (0.205, 0.301)	0.656 (0.636, 0.677)
Worry about COVID	0.042 (0.010, 0.074)		0.286*
Taken flu vaccine once	0.051 (0.027, 0.075)	0.032 (0.009, 0.053)	−0.001
Taken flu vaccine more than once	0.156 (0.127, 0.184)	0.085 (0.059, 0.109)	0.376*
Reliance on conservative media			−0.136*
Reliance on very conservative media		−0.045 (−0.071, −0.020)	−0.175*
Reliance on mainstream media		0.036 (0.012, 0.060)	0.256*
Reliance on social media			−0.165*
Strong Republican	0.038 (0.001, 0.074)		−0.108*
Not very strong Republican			−0.036
Closer to Republican			−0.051*
Closer to Democrat	−0.033 (−0.064, −0.001)		0.075*
Not very strong Democrat		0.030 (0.001, 0.053)	0.033
Strong Democrat	−0.078 (−0.118, −0.044)		0.123*
Female	−0.089 (−0.109, −0.067)		−0.157*
Age 25 y to 34 y	−0.063 (−0.087, −0.035)		−0.121*
Age 35 y to 44	−0.053 (−0.076, −0.029)		−0.095*
Age 45 y to 54 y	−0.050 (−0.072, −0.029)		−0.059*
Age 55 y to 64 y			0.027
Age 65+ y		0.080 (0.063, 0.098)	0.210*
Black identity	−0.062 (−0.091, −0.038)		−0.175*
Evangelical status		−*0.021 (*−*0.040,* −*0.002)*	−0.184*
Education level		*0.017 (0.002, 0.031)*	0.196*
Vaccination intention time 1		0.373 (0.342, 0.402)	
Variance explained	0.475	0.610	

Predictors that were fixed at zero in the model have no weight. Correlations with wave 1 intentions in column 3 were the observed values (with those at *P* < 0.01 noted with an asterisk), while correlations with latent variables were obtained from the measurement model for the SEM (*SI Appendix*, Table S1). Paths that only predicted within the 95% CI are italicized.

In terms of demographic differences at time 1, the results of the SEM indicated that those under age 55 y were less likely to intend to vaccinate than the reference group of 18- to 24-y-olds. In addition, those who identified as Black were less likely to intend to vaccinate than those who did not identify as Black (−0.062), and women were less likely than men, as well (−0.089). By time 2, unlike other variables that were associated with reluctance to vaccinate, Black individuals and women were no longer distinctly hesitant. Respondents ages 65 y and older reported the largest increases in vaccination intentions (0.080) between time 1 and time 2, while the intentions of those who identified as evangelicals decreased (−0.021).

Aside from demographic and political differences, the largest unique predictors at time 1 were general vaccination knowledge (0.408) and trust in health authorities (0.246). Not surprisingly, the latent variables representing these constructs were highly correlated (0.558) (*SI Appendix*, Table S1). Also highly related were COVID misinformation and belief in COVID-specific conspiracies (0.936), neither of which uniquely predicted vaccination intentions at time 1. In addition, trust was inversely related to acceptance of COVID-specific conspiracy beliefs (−0.722). However, prior flu vaccination behavior predicted time 1 vaccination intentions (0.156 and 0.051), as did worry about getting COVID-19 (0.042). Uses of various media were predictors of COVID-specific conspiracy thinking/beliefs, but not directly of vaccination intention at time 1. Although general conspiracy thinking/belief predicted COVID-specific thinking/beliefs, it was not uniquely related to vaccination intention at time 1 (Fig. 5).

The five significant predictors at time 1 (general vaccination knowledge, trust in health authorities, both measures of flu vaccination, and worry about getting COVID-19) carried over to time 2 vaccination intention by virtue of the positive relation between intentions at the two time points (0.373). Among these, everything except trust in health authorities uniquely predicted change between time 1 and time 2, indicating that they were continuing to have an effect. In addition, the use of extremely conservative media (−0.045) and mainstream media (0.036) were associated with changes over time, and belief in COVID-specific conspiracies emerged as a relatively strong disincentive at time 2 (−0.177). The conservative media effect is consistent with effects identified in earlier research (46). These predictors of change in intentions to vaccinate were robust to all controls.

Although misperceptions about the novel coronavirus (i.e., beliefs that the Centers for Disease Control and Prevention [CDC] admitted that many deaths were not really due to COVID-19, the COVID-19 death toll was overestimated, the flu shot could cause COVID, and masks don’t really help prevent the spread of COVID) were correlated with hesitancy at time 1 (Table 1, column 3), they did not emerge as distinct predictors in the SEM. Instead, these more localized beliefs shared variance with trust, conspiracy beliefs, and worry about the disease (*SI Appendix*, Table S1) and did not uniquely predict intentions at either time point. Hence, it seems unlikely that correcting these misperceptions alone would significantly increase vaccine uptake.

Respondents who identified as Democrats were always more prone to vaccinate than Republicans and political independents. This difference became more pronounced across the study’s time period. Specifically, Democrats’ intentions increased, while Republicans’ stayed largely steady (Fig. 3*B*).^†^ But, once we controlled for other variables in the SEM at time 1, partisanship did not appear to uniquely explain the persistent gap between partisans. In the presence of these controls, strong Republican identifiers were counterintuitively more likely to report that they intended to vaccinate than were independents (0.038; column 1 of Table 1), while strong Democrats were less likely to anticipate doing so (−0.078). Much of the variance associated with partisanship was shared with other predictors, such as the fact that Republicans were more likely to believe conspiracy theories and had lower trust in public health institutions than Democrats (*SI Appendix*, Table S3), as others have also found (47). When we examine changes between time 1 and time 2, partisanship displayed the expected effect. Additionally, among weak Democrats, intention to vaccinate increased from late September/early October to January/early February, all else being equal (0.030; column 2 of Table 1). Accordingly, to respond to our research question, the election outcome and prospective change in administration is a plausible but far from conclusive reason for the shift among weak Democrats. Whether the election shaped vaccination intentions by influencing other predictors in the model is also unclear.

## Discussion

Our findings underscore the central role that trust and knowledge play in increasing the likelihood of vaccinating. Trust in scientific institutions and spokespersons anchors time 1 vaccination intentions, and knowledge affects them at both times 1 and 2. Our trust finding underscores the importance of the National Academies of Sciences, Engineering, and Medicine (48) rapid expert consultation report recommendations that communities combat mistrust and build public confidence in COVID-19 vaccines by forming partnerships with community organizations; engaging with and centering the voices and perspectives of trusted messengers who have roots in the community; and engaging across multiple, accessible channels. Model engagement would include active listening and direct response to identified concerns using both traditional and nontraditional partners, including faith leaders (49).

Consistent with the prediction of our model, prior vaccination knowledge and behavior were among the predictors of change in vaccination intentions, with inaccurate beliefs about vaccines serving as a deterrent and getting flu shots in the past acting as a promoter. This evidence documents the need for the public health community to redouble its efforts to preemptively and persistently communicate not only about how vaccines in general work but also about their benefits, safety, and effectiveness. Doing so in the form of clear, transparent takeaways or gists would increase the memorability of the messaging (50). Our finding about the importance of knowledge about vaccination also underscores the importance of the 2021 policy changes announced by Facebook and Instagram (51), Twitter (52), and YouTube (53) barring misinformation about the safety, efficacy, or ingredients in currently authorized and administered vaccines. [The widespread dissemination of health misinformation on the platforms has been flagged by scholars (45, 54) and by a report by the US Surgeon General as a cause for concern (55).] At the same time, the importance of background vaccination knowledge is a reminder to the fact checking community that, in the process of focusing on COVID vaccine–specific misinformation, it should seek ways to address misbeliefs about vaccination more broadly. Our data also highlight the value of motivating yearly vaccination against seasonal flu.

Interestingly, trust in health authorities and worry were predictors of intent to vaccinate in late September/early October but not of positive change in willingness to vaccinate over time. This suggests that anxiety and such trust in authorities had already maximized their role in persuading individuals to accept vaccines, and their influence carried over in the strong relation between vaccination intention at baseline and the follow-up.

Contrary to our expectations, general conspiracy thinking/belief did not act as a negative predictor of changed willingness to vaccinate. However, continued belief in various coronavirus-related conspiracy theories negatively predicted likelihood to change vaccination intention, suggesting that these remained as barriers apart from the more general vaccination factors in our model.

The association between forms of media reliance and embrace of conspiracy beliefs indicates the importance of placing counterveiling provaccination messaging by trusted messengers in those media channels that, both before and during the COVID pandemic, harbored vaccine misinformation and seeded distrust of national public health messengers. The conspiracy findings underscore, as well, the need to identify and deploy messaging able to undercut health-related conspiracy beliefs as soon as they begin circulating.

A number of limitations are inherent in this work. Because the sample was drawn from five battleground states, it is not representative of the national population. Like all surveys, ours is limited by its reliance on self-reports. We cannot, as a result, confirm, for example, that those who said they had taken the flu vaccine in the past actually did so. Nor do we know that reported and actual media reliance coincide or whether those who were reluctant at time 1 and accepting of vaccination at time 2 actually took a COVID-19 vaccine. At the same time, our reliance on late September/early October and January/early February data to assess changed vaccination intentions means that factors other than the election and inauguration of Biden may account for the increased willingness of some Democrats to vaccinate. And, because our measure of general conspiracy thinking/beliefs occurred in wave 11 and did not precede the two key waves, it functions appropriately in the model only if we assume that such beliefs are stable and hence unvarying across the study, as is presumed by the construct itself.

## Conclusion

As the public health community asks how the disposition to accept a newly authorized vaccine can be increased before one is created in response to the next pandemic, this study suggests the importance of proactively increasing knowledge about vaccination in general while also debunking prevalent deceptions about it; deploying ongoing health interventions and messages bolstering trust in health and science authorities, especially those at the community level; counteracting misinformation in regard to conspiracy thinking/beliefs about the vaccine in question; and maximizing uptake of existing vaccines such as that for the seasonal flu.

## Materials and Methods

Data for the study come from a probability-based sample of Americans in five states interviewed as part of the 2020 Annenberg IOD Election Study. In the IOD Election Study, using address-based sampling, individuals were recruited by the survey firm SSRS to join the panel, and panelists were invited to answer surveys on an approximately monthly basis between April of 2020 and March of 2021. Between November of 2019 and March of 2020, 290,059 households were mailed letters recruiting members of those households to the study; 18,664 eligible respondents completed the recruitment survey either online or by telephone, reflecting a response rate of 7.0% (American Association for Public Opinion Research Response Rate 3; e = 0.92). Of these individuals, 15,490 were invited to complete the first wave to become panelists, and 11,686 did so (yielding a retention rate of 75.4% and a cumulative response rate of 5.3% [Cumulative Response Rate 3]; see ref. 56). Sampling for the study was conducted in four targeted states (Florida, Michigan, Pennsylvania, and Wisconsin) and five targeted areas (Pinellas County, FL; Macomb County, MI; Montgomery County, OH; Luzerne County, PA; and both Kenosha and Racine Counties, WI). These states and counties were chosen because they were viewed as particularly relevant to the Electoral College outcome in the 2020 US presidential election.^‡^ In each state and county, Census block groups with larger numbers of African American and Hispanic residents were sampled at disproportionate rates, yielding numbers that were close to statewide and countywide proportions after recruitment. After recruitment, the anesrake software in R (57) was used to weight all data to match benchmarks from the American Community Survey on sex, age group, education level, race/ethnicity, sex × age, sex × education, education × age, and region of the state (for statewide samples only).

Each wave was collected over the course of 3 wk (not always sequential) by inviting respondents from three groups that were randomly selected from the panelists just before the first substantive wave (replicates). Respondents were part of the same replicate for all waves. Recruitment was conducted between November 25, 2019 and April 21, 2020; wave 1 was conducted between April 29 and May 19, 2020; wave 2 was conducted between June 10 and June 30, 2020; wave 3 was conducted between July 1 and July 21, 2020; wave 4 was conducted between July 27 and August 16, 2020; wave 5 was conducted between August 28 and September 17, 2020; wave 6 was conducted between September 22 and October 14, 2020; wave 7 was conducted between October 16 and November 2, 2020; wave 8 was conducted between November 4 and November 24, 2020; wave 9 was conducted between December 2 and December 22, 2020; wave 10 was conducted between January 8 and February 3, 2021; and wave 11 was conducted for only the first replicate between February 16 and February 22, 2021. Although some respondents failed to complete any given wave, there was little attrition from the first wave through the rest of the study. Of the 10,243 respondents recruited at wave 1, 82.9% had complete vaccination intention data at both waves 6 and 10, with similar attrition at both waves (834 not participating in wave 6 and 898 not participating in wave 10).

The University of Pennsylvania’s Institutional Review Board (IRB) determined that the study met the criteria for the IRB review exemption authorized by 45 CFR 46.104, category #4,2.

### GAMs.

Trends in the vaccination tendencies of various groups were assessed using GAMs and were calculated using the mgcv software in R (58). The GAMs were used to generate spline smooths for each group of respondents over time. These models do not include any control variables and simply identify group-level differences in trends. Responses for continuous variables in Fig. 4 were divided into categories to reveal how these trends differed across subpopulations. With a single exception, response labels shown indicate the response option to scale questions that was most similar to each respondent’s score on the corresponding index (e.g., a mean trust score of 0.1 was coded as “Not at all” because it was closer to 0 than to 0.25 for “A little”). This was not possible for vaccination knowledge (Fig. 4*B*), where some items were reverse scored. Here, responses were divided, with those less than 0.45 coded as “Mostly misinformed,” those above 0.55 coded as “Mostly knowledgeable,” and those between the two coded as “About chance.” SEs in GAM plots represent 95% CIs. Predicted values for each variable in GAM plots were weighted to produce a population representative estimate within each wave and replicate.

The SEM examines factors that predict vaccination intentions in two survey waves: late September/early October of 2020, and January/early February of 2021 (for details and questions asked, see *SI Appendix*).

We used Mplus version 8.5 (59) to fit a path model (Fig. 5) that described the relation between predictors and vaccination intention at time 1 and the subsequent relations between those predictors and change in vaccination intention at time 2. As seen in the figure, predictors defined as latent variables are pictured as ovals, while observed scores, such as vaccination intention, are enclosed in rectangles. Predictors included all of the factors in the theoretical model in Fig. 1 along with various demographic and political affiliation variables. Our measure of general conspiracy thinking/beliefs was only acquired on one survey occasion, but Mplus imputed scores for this variable along with other missing scores using full information maximum likelihood estimation.

An initial test of the measurement model for the latent factors revealed excellent fit: comparative fit index (CFI) of 0.944, root-mean-squared estimate (RMSEA) of 0.060 (90% CI = 0.058, 0.061), and standardized mean standard residual (SRMR) of 0.048. The loadings for the latent factors are provided in *SI Appendix*, Table S1 along with the interfactor correlations. There was one residual correlation between the two items on the trust factor that referred to the CDC. Otherwise, all of the items loaded only on their respective factors. Correlations between the factors in the causal model and vaccination intention at time 1 are reported in *SI Appendix*.

Paths that are not pictured include the correlations between the predictors and the paths from the various demographic variables that were allowed to correlate with predictors of vaccination intention and with vaccination intention at both time 1 and time 2. Their path weights for vaccination are shown in Table 1. We used maximum likelihood estimates and bootstrapping with 1,000 samples to construct 99% CIs for all paths in the model. Those paths that survived with at least 90% CIs were retained in the final model. The final model provided an excellent fit to the data as indexed with a CFI = 0.920, RMSEA (90% CI) = 0.046 (0.045, 0.047), and SRMR = 0.033.

## Supplementary Material

Supplementary File

## Data Availability

Survey panel data have been deposited in Center for Open Science (DOI: https://doi.org/10.17605/OSF.IO/PW8CE) (60).

## Footnotes

*The solid green line treats respondents who reported that they had already vaccinated in the very likely category, whereas the dotted green line does not include these individuals. Hence, the difference between the two lines reflects the proportion of the population that was vaccinated between late December and late February. Shaded areas denote the periods of the two waves.

^†^Trends in this figure were generated by creating a separate spline for each partisanship category in a GAM predicting the intention to vaccinate, which was set to range from 0 (“Not at all likely”) to 1 (“Very likely”).

^‡^The states and counties had been won by Barack Obama in 2012 and by Donald Trump in 2016.

## Change History

January 21, 2022: The byline has been updated.
