# Supplementary material for: The role of non–COVID-specific and COVID-specific factors in predicting a shift in willingness to vaccinate: A panel study
Source: Proc Natl Acad Sci U S A. 2021 Dec 20;118(52):e2112266118. doi: 10.1073/pnas.2112266118 (PMC8719857; doi:10.1073/pnas.2112266118)
Supplement: Supplementary File [file pnas.2112266118.sapp.pdf]

PNAS
www.pnas.org

Supplementary Information for

The role of non-COVID-specific and COVID-specific factors in predicting a shift in willingness to vaccinate: A panel study

Kathleen Hall Jamieson

Daniel Romer

Patrick E. Jamieson

Kenneth M. Winneg

Josh Pasek

Corresponding author: Kathleen Hall Jamieson

Email: kathleen.jamieson@asc.upenn.edu

This PDF file includes:

Supplementary text - Measures

Figure S1

Table S1, S2, and S3

SI References

Measures

Vaccination History (wave 3)

Respondents in wave 3 were asked: "In the past two years, did you get a flu shot in both years, one year but not the other, or did you not get a flu shot in the past two years?" Response options were: "Got a flu shot both years," "Got a flu shot one year, but not the other," and "Did not get a flu shot in the past two years." Two dummy variables were created to identify those that had only one shot in the prior two years and those that had two shots.

Vaccination Knowledge (wave 3, 4 items, alpha=.78)

Respondents in wave 3 were asked four questions about their general beliefs about vaccines that were used to create a latent SEM factor.

Vaccines Safe. Respondents in wave 3 were asked: "How much do you agree or disagree with the following statement? Vaccines approved for use in the U.S. are safe." Response options were: "Strongly disagree" (coded: 0), "Somewhat disagree" (.25), "Neither agree nor disagree" (.5), "Somewhat agree" (.75), and "Strongly agree" (1).

Vaccine Immunity Better. Respondents in wave 3 were asked: "How much do you agree or disagree with the following statement? It is better to develop immunity by receiving a vaccine than by getting the disease." Response options were: "Strongly disagree" (coded: 0), "Somewhat disagree" (.25), "Neither agree nor disagree" (.5), "Somewhat agree" (.75), and "Strongly agree" (1).

MMR Autism. Respondents in wave 3 were asked: "Please indicate if you believe the statement below is true, false, or if you aren't sure. Vaccines given to children for diseases like measles, mumps, and rubella **do not** cause autism." Response options were: "Definitely false" (coded: 0), "Probably false" (.33), "Probably True" (.67), "Definitely true" (1) and "Not Sure" (.5).

Vaccine Toxins. Respondents in wave 3 were asked: "Please indicate if you believe the statement below is true, false, or if you aren't sure. Vaccines in general are full of toxins and harmful ingredients like 'antifreeze'." Response options were: "Definitely false" (coded: 1), "Probably false" (.67), "Probably True" (.33), "Definitely true" (0) and "Not Sure" (.5).

Trust in Health Authorities (wave 5, 3 items, alpha=.86)

Respondents in wave 5 were asked three questions about their trust in public health authorities that were used to create a latent SEM factor.

Trust CDC Motivations. Respondents in wave 5 were asked: "How much, if at all, do you trust the leaders of institutions such as the U.S. Centers for Disease Control and Prevention (CDC) and the National Institutes of Health (NIH) to act in the best interest of people like you?" Response options were: "A great deal" (coded: 1), "A lot" (.75), "A moderate amount" (.5), "A little" (.25), and "Not at all" (0).

Trust Fauci. Respondents in wave 5 were asked: “How much, if at all, do you trust what they tell you about the coronavirus pandemic: Dr. Anthony Fauci of the National Institutes of Health (NIH)” Response options were: “A great deal” (coded: 1), “A lot” (.75), “A moderate amount” (.5), “A little” (.25), and “Not at all” (0).

Trust CDC. Respondents in wave 5 were asked: “How much, if at all, do you trust what they tell you about the coronavirus pandemic: U.S. Centers for Disease Control and Prevention (CDC)?” Response options were: “A great deal” (coded: 1), “A lot” (.75), “A moderate amount” (.5), “A little” (.25), and “Not at all” (0).

Reliance on Very Conservative Media (wave 9)

Respondents in wave 9 were asked: “How often, if at all, do you get information about the most important issues of the day from **Sources such as Newsmax, One America News (OAN), Gateway Pundit, or Parler?** Response options were: “Never” (coded: 0), “Rarely” (.25), “Sometimes” (.5), “Often” (.75), or “All the time” (1).

Reliance on Conservative Media (multiple waves, 5 items, alpha=.92)

Respondents across four waves were asked questions about their use of conservative media that were averaged together to make an index.

Respondents in wave 1 were asked: “How often, if at all, do you get information about the coronavirus pandemic from **Sources such as Fox News Channel or Rush Limbaugh?** Response options were: “Never” (coded: 0), “Rarely” (.25), “Sometimes” (.5), “Often” (.75), or “All the time” (1).

Respondents in wave 2 were asked: “How often, if at all, do you get information about the most important issues of the day from **Sources such as Fox News Channel or Rush Limbaugh?** Response options were: “Never” (coded: 0), “Rarely” (.25), “Sometimes” (.5), “Often” (.75), or “All the time” (1).

Respondents in wave 5 were asked: “How often, if at all, do you get information about the 2020 U.S. Presidential election from **Sources such as Fox News Channel or Rush Limbaugh?** Response options were: “Never” (coded: 0), “Rarely” (.25), “Sometimes” (.5), “Often” (.75), or “All the time” (1).

Respondents in wave 6 were asked: “How much information do you get from each of the following sources? Use a scale from 0 to 5, where 0 means you get ‘No information’ from these sources, and 5 means you get ‘A lot of information’ from these sources. Of course, you can use any number between 0 and 5. **Sources such as Fox News, Rush Limbaugh, Breitbart News, One America News, or The Drudge Report.**” Response options were: 0 (coded: 0), 1 (.2), 2 (.4), 3 (.6), 4 (.8), and 5 (1).

Respondents in wave 9 were asked: “How often, if at all, do you get information about the most important issues of the day from **Sources such as Fox News Channel or Rush Limbaugh?** Response options were: “Never” (coded: 0), “Rarely” (.25), “Sometimes” (.5), “Often” (.75), or “All the time” (1).

Reliance on Mainstream Media (multiple waves, 13 items)

Respondents across waves were asked questions about their use of four different categories of mainstream media use that were each averaged together to make four separate indexes. For each respondent the mainstream category that solicited the most use across time points was treated as their estimate of mainstream use.

Popular Short Form Broadcast/Journalism Use (4 items, alpha=.84)

Respondents in wave 1 were asked: "How often, if at all, do you get information about the coronavirus pandemic from **Sources such as ABC News or USA Today?** Response options were: "Never" (coded: 0), "Rarely" (.25), "Sometimes" (.5), "Often" (.75), or "All the time" (1).

Respondents in wave 2 were asked: "How often, if at all, do you get information about the most important issues of the day from **Sources such as ABC News or USA Today?** Response options were: "Never" (coded: 0), "Rarely" (.25), "Sometimes" (.5), "Often" (.75), or "All the time" (1).

Respondents in wave 5 were asked: "How often, if at all, do you get information about the 2020 U.S. Presidential election from **Sources such as ABC News or USA Today?** Response options were: "Never" (coded: 0), "Rarely" (.25), "Sometimes" (.5), "Often" (.75), or "All the time" (1).

Respondents in wave 6 were asked: "How much information do you get from each of the following sources? Use a scale from 0 to 5, where 0 means you get 'No information' from these sources, and 5 means you get 'A lot of information' from these sources. Of course, you can use any number between 0 and 5. **Sources such as ABC News, CBS News, or NBC News.**" Response options were: 0 (coded: 0), 1 (.2), 2 (.4), 3 (.6), 4 (.8), and 5 (1).

Legacy Print Media Use (4 items, alpha=.90)

Respondents in wave 1 were asked: "How often, if at all, do you get information about the coronavirus pandemic from **Sources such as The New York Times or The Washington Post?** Response options were: "Never" (coded: 0), "Rarely" (.25), "Sometimes" (.5), "Often" (.75), or "All the time" (1).

Respondents in wave 2 were asked: "How often, if at all, do you get information about the most important issues of the day from **Sources such as The New York Times or The Washington Post?** Response options were: "Never" (coded: 0), "Rarely" (.25), "Sometimes" (.5), "Often" (.75), or "All the time" (1).

Respondents in wave 5 were asked: "How often, if at all, do you get information about the 2020 U.S. Presidential election from **Sources such as The New York Times or The Washington Post?** Response options were: "Never" (coded: 0), "Rarely" (.25), "Sometimes" (.5), "Often" (.75), or "All the time" (1).

Respondents in wave 6 were asked: "How much information do you get from each of the following sources? Use a scale from 0 to 5, where 0 means you get 'No information' from these

sources, and 5 means you get 'A lot of information' from these sources. Of course, you can use any number between 0 and 5. **Sources such as Associated Press, The New York Times, the Washington Post, or the Wall Street Journal.**" Response options were: 0 (coded: 0), 1 (.2), 2 (.4), 3 (.6), 4 (.8), and 5 (1).

CNN Use (3 items, alpha=.89)

Respondents in wave 1 were asked: "How often, if at all, do you get information about the coronavirus pandemic from **CNN?** Response options were: "Never" (coded: 0), "Rarely" (.25), "Sometimes" (.5), "Often" (.75), or "All the time" (1).

Respondents in wave 2 were asked: "How often, if at all, do you get information about the most important issues of the day from **CNN?** Response options were: "Never" (coded: 0), "Rarely" (.25), "Sometimes" (.5), "Often" (.75), or "All the time" (1).

Respondents in wave 5 were asked: "How often, if at all, do you get information about the 2020 U.S. Presidential election from **CNN?** Response options were: "Never" (coded: 0), "Rarely" (.25), "Sometimes" (.5), "Often" (.75), or "All the time" (1).

Public Broadcasting Use (2 items, alpha=.84)

Respondents in wave 2 were asked: "How often, if at all, do you get information about the most important issues of the day from **Sources such as National Public Radio (NPR), PBS Newshour, or Washington Week?** Response options were: "Never" (coded: 0), "Rarely" (.25), "Sometimes" (.5), "Often" (.75), or "All the time" (1).

Respondents in wave 5 were asked: "How often, if at all, do you get information about the 2020 U.S. Presidential election from **Sources such as National Public Radio (NPR), PBS Newshour, or Washington Week?** Response options were: "Never" (coded: 0), "Rarely" (.25), "Sometimes" (.5), "Often" (.75), or "All the time" (1).

Reliance on Social Media (multiple waves, 4 items)

Social Media Use (4 items, alpha=.89)

Respondents in wave 1 were asked: "How often, if at all, do you get information about the coronavirus pandemic from **social media such as Facebook or Twitter?** Response options were: "Never" (coded: 0), "Rarely" (.25), "Sometimes" (.5), "Often" (.75), or "All the time" (1).

Respondents in wave 2 were asked: "How often, if at all, do you get information about the most important issues of the day from **social media such as Facebook or Twitter?** Response options were: "Never" (coded: 0), "Rarely" (.25), "Sometimes" (.5), "Often" (.75), or "All the time" (1).

Respondents in wave 5 were asked: "How often, if at all, do you get information about the 2020 U.S. Presidential election from **social media such as Facebook or Twitter?** Response options were: "Never" (coded: 0), "Rarely" (.25), "Sometimes" (.5), "Often" (.75), or "All the time" (1).

Respondents in wave 6 were asked: "How much information do you get from each of the following sources? Use a scale from 0 to 5, where 0 means you get 'No information' from these sources, and 5 means you get 'A lot of information' from these sources. Of course, you can use any number between 0 and 5. **Sources such as Facebook, Twitter, or YouTube?** Response options were: 0 (coded: 0), 1 (.2), 2 (.4), 3 (.6), 4 (.8), and 5 (1).

General Conspiracy Thinking/Beliefs (wave 11, 3 items, alpha=.76)

To capture conspiracy thinking in the form of general conspiracy thinking/beliefs, respondents in wave 11 were asked three questions from a scale developed and validated by Uscinski et al. and used in their prior COVID research (1). We used the items to create a latent SEM factor.

Respondents in wave 11 were asked: "Much of our lives is controlled by plots hatched in secret places." Response options were: "Strongly disagree" (coded: 0), "Somewhat disagree" (.25), "Neither agree nor disagree" (.5), "Somewhat agree" (.75), and "Strongly agree" (1).

Respondents in wave 11 were asked: "Even though we live in a democracy, a few people will always run things anyway." Response options were: "Strongly disagree" (coded: 0), "Somewhat disagree" (.25), "Neither agree nor disagree" (.5), "Somewhat agree" (.75), and "Strongly agree" (1).

Respondents in wave 11 were asked: "The people who really 'run' the country are not known to the voters." Response options were: "Strongly disagree" (coded: 0), "Somewhat disagree" (.25), "Neither agree nor disagree" (.5), "Somewhat agree" (.75), and "Strongly agree" (1).

Worry About COVID-19 (wave 5)

Respondents in wave 5 were asked: "How worried, if at all, are you that the health of someone in your family will be seriously affected from getting the coronavirus?" Response options were: "Not at all worried" (coded: 0), "Not too worried" (.33), "Somewhat worried" (.66), "Very worried" (1), and "This has already happened" (set to missing).[1]

COVID-19 Specific Conspiracy Thinking/Beliefs (wave 6, 3 items, alpha=.77)

Respondents in wave 6 were asked three questions about their beliefs in coronavirus-specific conspiracy theories that were used to create a latent SEM factor.

CDC Exaggerated. Respondents in wave 6 were asked: "Please indicate if you believe the statement below is true, false, or if you aren't sure. Some health officials at the U.S. Centers for Disease Control and Prevention, also known as the CDC, have exaggerated the danger posed by the coronavirus in order to damage the Trump presidency." Response options were:

[1] Respondents who said that this had already happened may have held varying levels of worry prior to these health effects. Because maximum likelihood imputation was used in the structural equation model, we decided that imputing for these individuals would yield a more valid estimate of their worry than treating the category as if it corresponded with a particular level of worry.

“Definitely false” (coded: 0), “Probably false” (.33), “Probably True” (.67), “Definitely true” (1) and “Not Sure” (.5).

FDA Delayed. Respondents in wave 6 were asked: “Please indicate if you believe the statement below is true, false, or if you aren't sure. Health officials at the Food and Drug Administration, also known as the FDA, who oppose Donald Trump's re-election are delaying the approval of COVID-19 treatments until after the election.” Response options were: “Definitely false” (coded: 0), “Probably false” (.33), “Probably True” (.67, “Definitely true” (1) and “Not Sure” (.5).

Pharma Created. Respondents in wave 6 were asked: “Please indicate if you believe the statement below is true, false, or if you aren't sure. The pharmaceutical industry created the coronavirus to increase sales of its drugs and vaccines.” Response options were: “Definitely false” (coded: 0), “Probably false” (.33), “Probably True” (.67) , “Definitely true” (1) and “Not Sure” (.5).

COVID-19 Specific Knowledge/Misinformation (wave 6, 3 items, alpha=.69)

Respondents in wave 6 were asked three questions about their beliefs in coronavirus-specific misperceptions that did not involve a conspiracy that were used to create a latent SEM factor.

CDC Admitted. Respondents in wave 6 were asked: “Please indicate if you believe the statement below is true, false, or if you aren't sure. The U.S. Centers for Disease Control and Prevention, also known as the CDC, has admitted that most of the deaths attributed to COVID-19 were actually caused by other serious illnesses and not by the coronavirus.” Response options were: “Definitely false” (coded: 0), “Probably false” (.33), “Probably True” (.67) , “Definitely true” (1) and “Not Sure” (.5).

COVID Overestimated. Respondents in wave 6 were asked: “Do you believe that the **actual** number of Americans who have died from COVID-19 is higher, lower, or about the same as the **reported** number of deaths from COVID-19?” Response options were: “Higher” (coded: 0), “Lower” (1), and “About the Same” (0).

Mask Increases Risk. Respondents in wave 6 were asked: “Do you believe that wearing a mask or face covering increases, decreases, or has no effect on your chances of contracting COVID-19?” Response options were: “Increases” (coded: 1), “Decreases” (0), and “No Effect” (.5).

Intention to Vaccinate for COVID-19 (multiple waves)

Respondents in wave 3 were asked: “If a vaccine that protects people from the coronavirus, also known as COVID-19, becomes available, how likely, if at all, would you be to get vaccinated?” Response options were: “Not at all likely to get vaccinated” (coded: 0), “Not too likely” (.33), “Somewhat likely” (.67), and “Very likely to get vaccinated” (1).

Respondents in waves 5-9 were asked: “If a no-cost vaccine that protects people from the coronavirus, also known as COVID-19, becomes available and is approved by the Food and Drug Administration, also known as the FDA, how likely, if at all, would you be to get vaccinated?” Response options were: “Not at all likely to get vaccinated” (coded: 0), “Not too likely” (.33), “Somewhat likely” (.67), and “Very likely to get vaccinated” (1).

Respondents in waves 10 and 11 who said that they had not already been vaccinated were asked: “When a no-cost vaccine that is approved by the Food and Drug Administration, also known as the FDA, to protect people from the coronavirus, also known as COVID-19, becomes available to you, how likely, if at all, would you be to get vaccinated?” Response options were: “Not at all likely to get vaccinated” (coded: 0), “Not too likely” (.33), “Somewhat likely” (.67), and “Very likely to get vaccinated” (1).

Control Variables

The following control variables were included in the model and the data were collected during the recruitment phase of the study:

Partisanship

Partisanship dummy variables were created by combining three variables, all asked in the recruitment wave.

PID1. Respondents at recruitment were asked: “Do you consider yourself a Democrat, a Republican, an Independent, a supporter of some other party, or none of these?” Response options were “Democrat,” “Republican,” “Independent,” “Other,” and “None of these.”

PID2. Respondents who answered either “Democrat” or “Republican” to PID1 were asked: “Would you call yourself a strong (Democrat/Republican) or not a very strong (Democrat/Republican)?” Response options were “Strong,” and “Not very strong.”

PID3. Respondents who answered “Independent,” “Other,” or “None of these” to PID1 were asked: “Do you think of yourself as closer to the Democratic Party or the Republican Party?” Response options were “Closer to the Democratic Party,” “Closer to the Republican Party,” and “Not closer to either one.”

Responses were combined and recoded into seven dummy variables for: “Strong Republican” (PID1=Republican & PID2=Strong), “Not very strong Republican” (PID1=Republican & PID2=Not very strong), “Closer to the Republican Party” (PID3=Closer to the Republican Party), “Independent” (PID3= Not closer to either one), “Closer to the Democratic Party” (PID3=Closer to the Democratic Party), “Not very strong Democrat” (PID1=Democrat & PID2=Not very strong), and “Strong Democrat” (PID1=Democrat & PID2=Strong),.

Sex

Respondents at recruitment were asked: "Which of the following best describes your gender identity?" Response options were: "Male," "Female," "Transgender or non-binary," "None of these." Dummy variables were created to identify respondents who answered "Male" and "Female." (note: there were too few respondents in the other categories to include them in the generalized additive model plots)

Age

Respondents at recruitment were asked: "In what year were you born?" Responses were recoded into the following categories: "18 to 24," "25 to 34," "35 to 44," "45 to 54," "55 to 64," and "65 and older."

Race/Ethnicity

The Race/Ethnicity variable was created by combining responses to two variables.

Hispanic Identification. Respondents at recruitment were asked: Are you of Hispanic, Latino, or Spanish origin or descent?" Response options were: "Yes," and "No."

Race. Respondents at recruitment were asked: "How would you describe your race?" Response options were: "White," "Black or African American," "Asian," "Native American," "Native Hawaiian or Pacific Islander," "Some other race (specify)," or "Mixed Race (Specify)." (Note: respondents could only choose one of these options).

Four dummy variables were created by combining responses to these questions. Respondents were coded as *White, non-Hispanic* if they answered "No" to the Hispanic question and "White" to the Race variable. Respondents were coded as *Black, non-Hispanic* if they answered "No" to the Hispanic question and "Black or African American" to the Race variable. Respondents were coded as Other/Mixed*, non-Hispanic* if they answered "No" to the Hispanic question and "Other" or "Mixed race" to the Race variable. Respondents were coded *Hispanic* if they answered "Yes" to the Hispanic question regardless of their answer to the Race question.

Evangelical Status

Religion. Respondents at recruitment were first asked: "What is your present religion, if any?" Response options were: "Protestant," "Catholic," "Mormon," "Orthodox," "Another type of Christian," "Jewish," "Muslim," "Some other religion," or "Atheist, Agnostic, or No religion."

Evangelical status. Respondents who answered the religion question saying they were "Protestant", "Catholic", "Mormon", "Orthodox" or "Another type of Christian" were asked: "Do you consider yourself an evangelical or born-again Christian, or not?" Response options were: "Yes, evangelical or born-again Christian" or "No, not evangelical or born-again

Christian." Respondents who provided other answers to the religion question were coded as "No, not evangelical or born-again Christian."

Education Level (4 category, recoded as linear)

All respondents were asked: What is the highest level of education you have completed or the highest degree you have received? Response options were: "Less than high school (Grades 1-8 or no formal schooling)," "High school incomplete (Grades 9-11 or Grade 12 with NO diploma)," "High school graduate (Grade 12 with diploma or GED certificate)," "Some college, no degree (includes some community college)," "Two-year associate degree from a college or university," "Four-year college or university degree/Bachelor's degree (e.g., BS, BA, AB)," "Some postgraduate or professional schooling, no postgraduate degree (e.g. some graduate school)," "Postgraduate or professional degree, including master's, doctorate, medical or law degree (e.g., MA, MS, PhD, MD, JD, graduate school)."

Responses were combined and recoded as a linear variable with the following categories: "High school graduate or less" (coded: 0), "Some college" (.33), "College graduate" (.67), "Post-graduate degree" (1).

Additional Trends Over Time

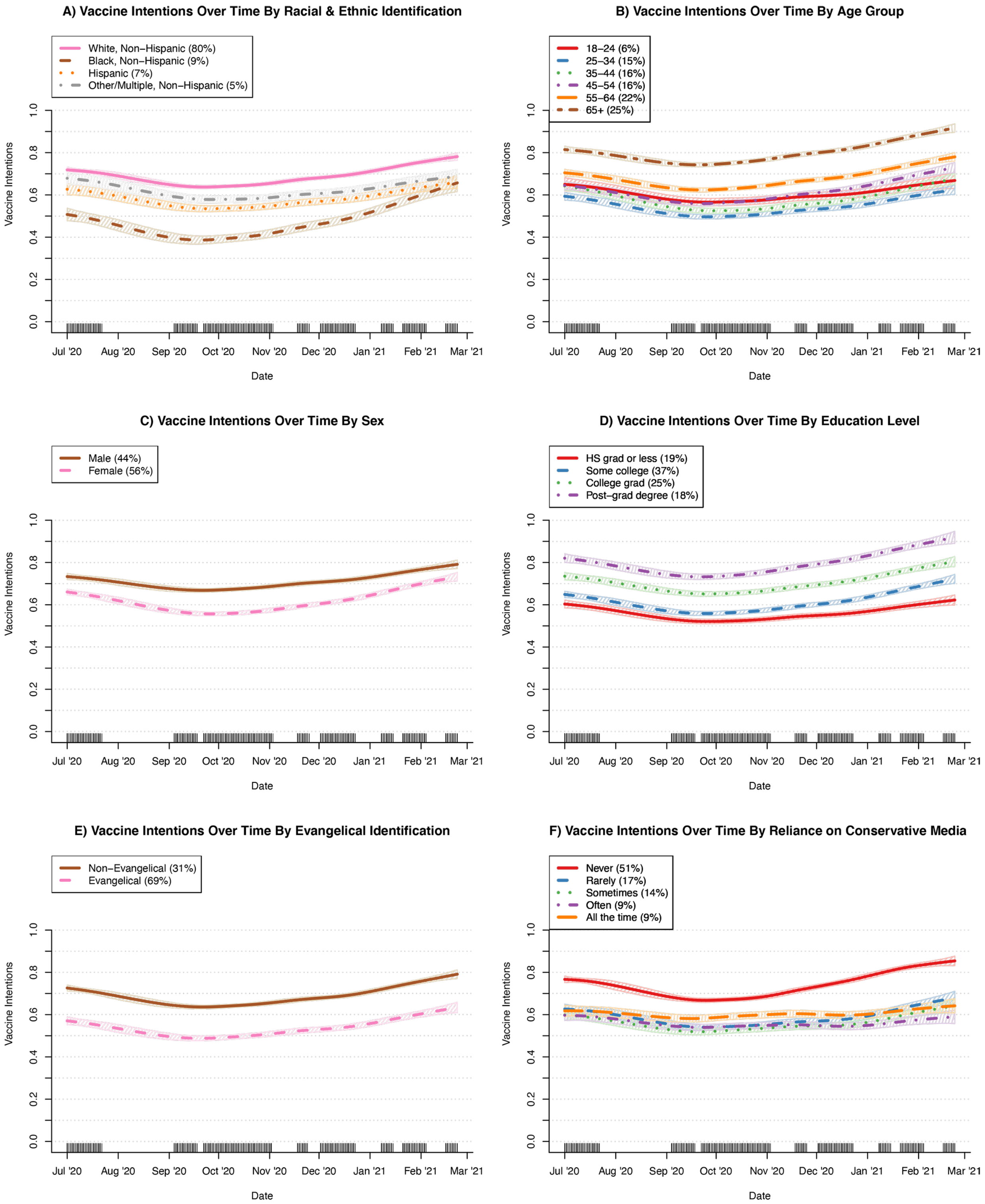
A) Vaccine Intentions Over Time By Racial & Ethnic Identification
White, Non–Hispanic (80%)
Black, Non–Hispanic (9%)
Hispanic (7%)
Other/Multiple, Non–Hispanic (5%)
B) Vaccine Intentions Over Time By Age Group
18–24 (6%)
25–34 (15%)
35–44 (16%)
45–54 (16%)
55–64 (22%)
65+ (25%)
C) Vaccine Intentions Over Time By Sex
Male (44%)
Female (56%)
D) Vaccine Intentions Over Time By Education Level
HS grad or less (19%)
Some college (37%)
College grad (25%)
Post–grad degree (18%)
E) Vaccine Intentions Over Time By Evangelical Identification
Non–Evangelical (31%)
Evangelical (69%)
F) Vaccine Intentions Over Time By Reliance on Conservative Media
Never (51%)
Rarely (17%)
Sometimes (14%)
Often (9%)
All the time (9%)
Vaccine Intentions
0.0
0.2
0.4
0.6
0.8
1.0
Jul '20
Aug '20
Sep '20
Oct '20
Nov '20
Dec '20
Jan '21
Feb '21
Mar '21
Date

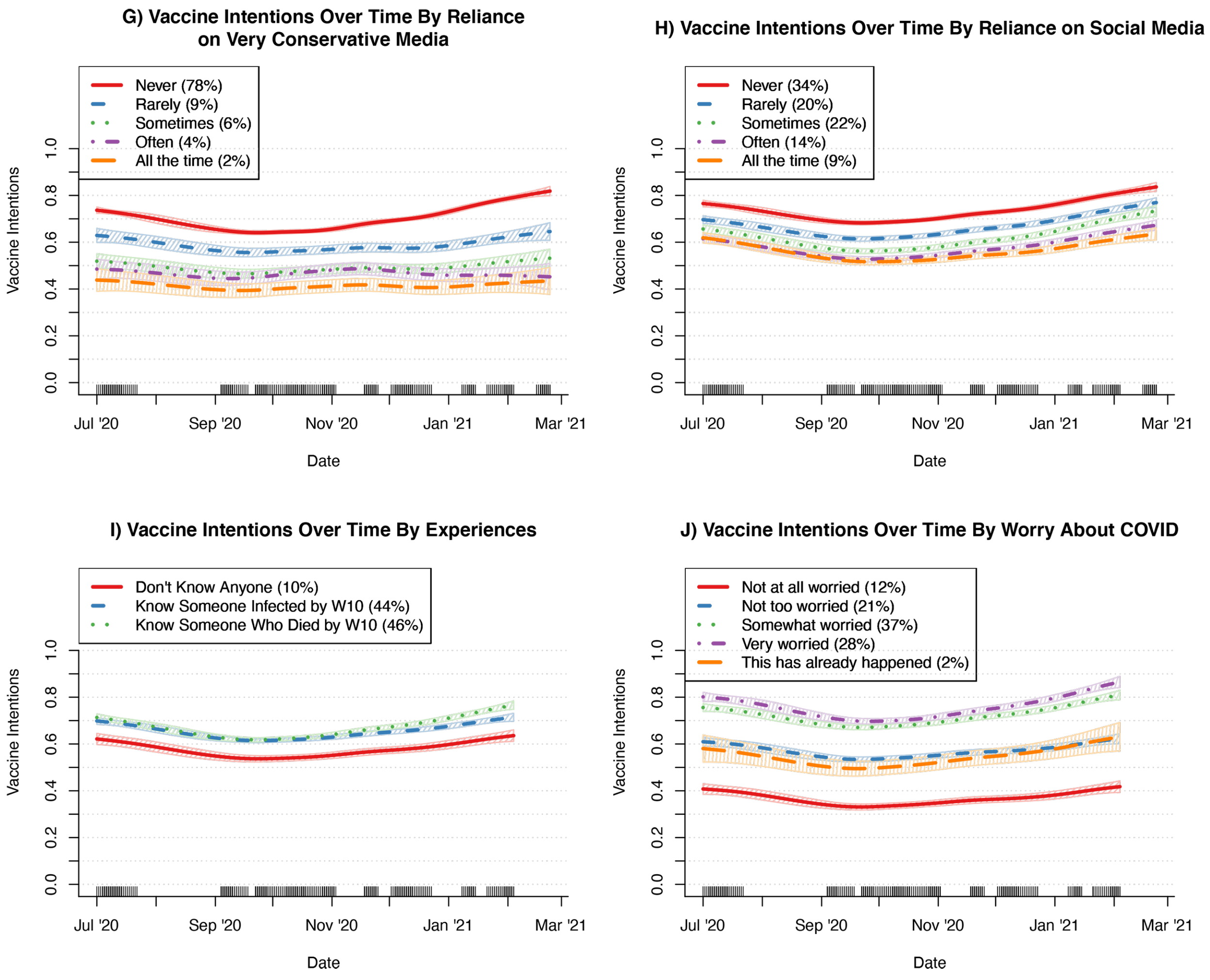

Figure S1 - Trends in vaccination intentions by additional covariates based on generalized additive models.

Table S1. Measurement Model Standardized Factor Loadings and Inter-Correlations

Factor	Factor Loading	Correlation With Trust	Correlation With Vaccine Knowledge	Correlation With Covid Specific Knowledge	Correlation With General Conspiracy Beliefs
Trust in Health Authorities					
CDC Motives	.622				
Fauci	.989				
CDC Information	.655				
Vaccine Knowledge		.558			
Safe	.794				
Immunity	.705				
Autism	.556				
Toxic	-.716				
Covid Specific Knowledge		-.754	-.583		
CDC admitted	.770				
Overestimated	.683				
Mask risk	-.555				
General Conspiracy Beliefs		-.565	-.556	.659	
Plots	.780				
Run things	.580				
Unknown	.776				
Covid Specific Conspiracy Beliefs		-.722	-.610	.936	.684
CDC Exaggerated	.878				
FDA Delayed	.526				
Pharma Created	.803				

Table S2 - Changes in Vaccination Intentions
Between Waves 6 and 10 (weighted)

		Wave 10 Response					
		Not at all likely	Not too likely	Somewhat likely	Very likely	Already vaccinated	Total
Wave 6 Response	Not at all likely	939.2 (11.1%)	370.8 (4.4%)	166.0 (2.0%)	109.2 (1.3%)	34.6 (0.4%)	1619.9 (19.1%)
	Not too likely	291.1 (3.4%)	377.2 (4.4%)	365.5 (4.3%)	311.1 (3.7%)	71.1 (0.8%)	1416.0 (16.7%)
	Somewhat likely	67.2 (0.8%)	240.6 (2.8%)	631.4 (7.4%)	1216.4 (14.3%)	211.2 (2.5%)	2366.8 (27.9%)
	Very likely	49.8 (0.6%)	72.7 (0.9%)	256.7 (3.0%)	2308.4 (27.2%)	405.8 (4.8%)	3093.3 (36.4%)
	Total	1347.3 (15.9%)	1061.3 (12.5%)	1419.6 (16.7%)	3945.1 (46.4%)	722.7 (8.5%)	8496 (100%)

Note: Cells reflect the weighted number and percentage of respondents shifting between each response in waves 6 and 10.

Table S3. Correlations between partisan predictors and vaccination beliefs in SEM.

Vaccination Belief	Partisan Predictor					
	Strong Republican	Not very Strong Republican	Closer to Republican	Closer to Democrat	Not very strong Democrat	Strong Democrat
Trust in Health Authorities	-.460	-.136	-.194	.193	.113	.456
Worry about Covid	-.290	-.082	-.141	.116	.074	.271
Covid Specific Misinformation						
Covid Specific Conspiracy Beliefs	.121	.124	.050	-.100	-.082	-.111
General Conspiracy Beliefs	.340	.096	.155	-.150	-.068	-.362
Vaccination Knowledge	-.201	-.036	-.073	.115	.037	.220

Note: Empty cells represent correlations that were fixed to zero due to lack of unique relationship with vaccination intention. All others fell within 99% CI.

SI References

1. J. E. Uscinski, *et al.*, Why do people believe COVID-19 conspiracy theories? *Harv. Kennedy Sch. Misinformation Rev.* **1** (2020).
